# Endoscopic Repair of CSF Rhinorrhea: An Institutional Experience

**Published:** 2016-01

**Authors:** Sarita Kumari Mishra, George Ani Mathew, Roshna Rose Paul, Syed Kamran Asif, Mary John, Ajoy Mathew Varghese, Mary Kurien

**Affiliations:** 1 *Department of Otorhinolaryngology,Indira Gandhi Institute of Medical Sciences, Patna, Bihar, India.*; 2 *Department of Otorhinolaryngology, Christian Medical College, Vellore, Tamilnadu, India.*

**Keywords:** Cribriform plate, CSF rhinorrhea, Transnasal Endoscopic Surgery, Subarachnoid Space, Valsalva Maneuver

## Abstract

**Introduction:**

Endoscopic repair is considered the treatment of choice in cerebrospinal fluid (CSF) rhinorrhea. The aim of our study was to analyze the etiopathogenesis of CSF rhinorrhea, the outcome of treatment and the causes of failure in a developing-country setting.

**Materials and Methods:**

A retrospective review of patients treated with endoscopic repair for CSF rhinorrhea at a tertiary care hospital in southern India from January 2002 to December 2009 identified 36 patients, the majority of them being women. The defects were closed in three layers using fat, fascia lata and nasal mucosa along with a fibrin sealant in the majority of the patients. Per-operatively, a subarachnoid drain was placed in all patients. Patients were followed up for 1 year.

**Results:**

Spontaneous onset of CSF rhinorrhea was noted in 61% of patients. The most common site of leak was found to be the left cribriform plate area. Hence the most common cause of CSF rhinorrhea in our study was spontaneous and the second most common was post-traumatic. Our success rate on the first attempt at endoscopic repair was 100%, with a recurrence rate of 6%. A large defect, failure of localization of the defect, or other co-morbid conditions such as chronic cough may be the most likely causes of recurrence of leak.

**Conclusion:**

Accurate localization of the site of lesion using a high-resolution computed tomography (CT) scan with magnetic resonance imaging (MRI) and confirmation of the site of leak by intraoperative Valsalva maneuver along with multilayered closure of the dural defect and post-operative lumbar drain appear to be essential for the successful endoscopic repair of CSF rhinorrhea.

## Introduction

Cerebrospinal fluid (CSF) rhinorrhea has been reported to be spontaneous or secondary to head trauma, surgery, neoplastic invasion, congenital malfor- mations; the most common causes being post-traumatic according to some authors and iatrogenic according to others (1,2). The risk of meningitis with untreated CSF rhinorrhea has also been reported to range from 23% to 60% (1,3,4). Since Wigand first reported the endoscopic repair of CSF rhinorrhea in 1981, it is now considered the preferred choice of treatment (3).

A retrospective study of patients who presented in our unit with CSF rhinorrhea and underwent endoscopic repair was performed. Etiopathogenesis, outcome of treatment and causes of failure were noted. Our experience is presented in this article. 

## Materials and Methods

A retrospective inpatient chart audit was performed for all cases of CSF leak repairs carried out in the ear, nose and throat (ENT) unit at Christian Medical College and Vellore Hospital from January 2002 to December 2009. 

**Fig1:**
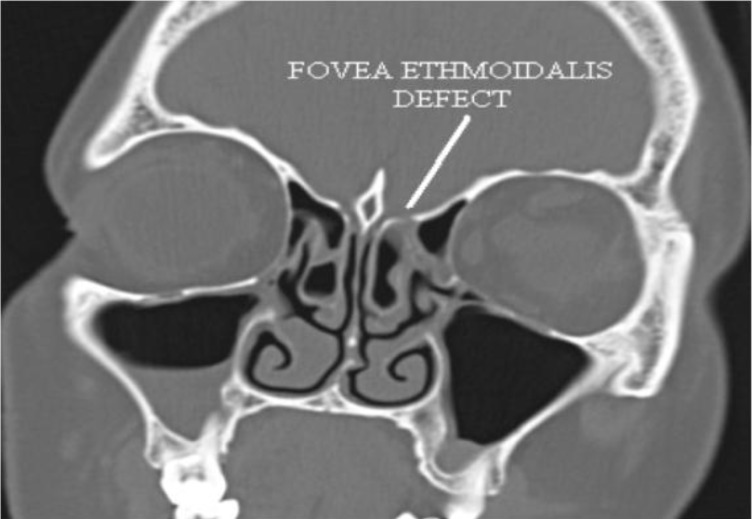
Defect in fovea ethmoidalis area

All cases were subjected to a detailed history, regarding the duration of leak, mode of onset, etiology and history of meningitis. Following this, diagnostic nasal endoscopy was performed to confirm the side of leak and to rule out other nasal pathologies. Nasal endoscopy was not useful in identifying the exact site of the leak. Nasal secretion was collected and sent for glucose concentration analysis to explore the likelihood of CSF rhinorrhea. All patients had a high-resolution computed tomography (CT) scan (Fig.1), with additional magnetic resonance imaging (MRI) cuts for identification of the site of leak and to search for meningocele and meningoencephalocele. Beta-2 transferrin in the nasal secretions was not assessed and intrathecal fluorescein injection was not used. All patients were vaccinated against pneumococcus. Patients who had meningitis were treated with crystalline penicillin in anti-meningitis doses for 3 weeks prior to surgery. Patients with chronic cough had a pulmonary medicine consultation for treatment prior to surgery. 

**Fig 2:**
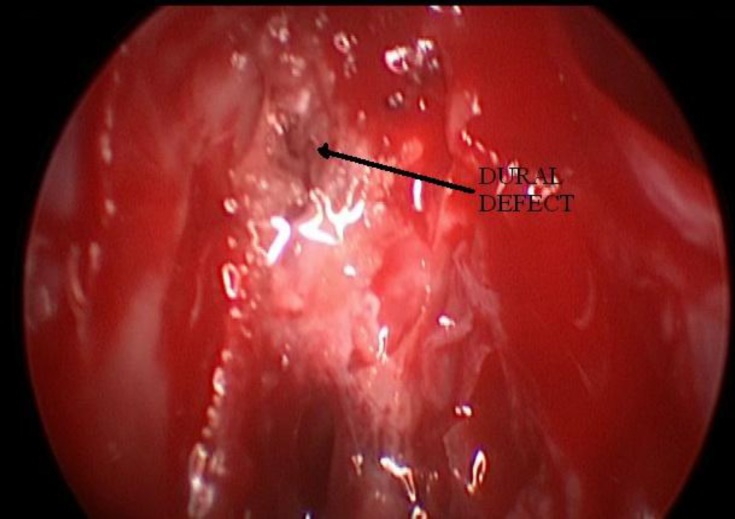
Endoscopic view of the dural defect

Patients were subjected to hypotensive general anesthesia. A lumbar drain was inserted and kept closed until the site of leak was identified. Fat and fascia lata were then harvested from the lateral side of the thigh. All operations were performed using standard endoscopic sinus surgery equipment without computer-assisted surgical navigation. The middle turbinate was excised and the suspected site of leak in the skull base was denuded of mucosa. The bulla ethmoidalis was opened in cases where the defect was found to be extending into the fovea ethmoidalis. The site of the leak was localized. In a few patients where an active leak was not seen, it was noted by Valsalva maneuver. The margins of the bony defect were then delineated (Fig.2).

At this stage, the lumbar drain was opened and fat was then inserted into the defect. The fascia lata was placed over the defect and a prepared fibrin sealant consisting of lyophilized vapor heated sealer human protein concentrate (TISSEEL) was injected onto the edges of the fascia. The mucosa from the middle turbinate was then spread over it, and the remaining fibrin sealant was again injected around the edge of the harvested middle turbinate mucosa. In two patients, a piece of the bone from the excised middle turbinate was used to seal the defect in addition to the fascia. Surgicel was then spread over the fascia/mucosa layer, and finally a Bismuth Iodoform Paraffin Paste pack was placed in position.

Postoperatively, patients were advised to adhere to strict bed rest. They were also given TED stockings and subcutaneous low molecular weight heparin for 5 days postoperatively to prevent deep vein thrombosis. Stool softeners were also given.A lumbar drain and nasal pack were removed on the 5^th^ and 7^th^ post-operative day, respectively. Thigh sutures were removed on the 10th day. An endoscopic inspection of the operative site with removal of crusts was performed on the same day, leaving the operative site untouched. All patients were followed up for 6–12 months. 

## Results

A total of 36 patients were identified in whom endoscopic CSF rhinorrhea repair was performed from January 2002 to December 2009. One patient had an additional external approach for frontal sinus CSF leak repair. Most patients were in their 30s, with the youngest being 8 months old and the oldest being 64 years. Twenty-eight of the subjects were women, and eight were men.

Fifteen of the 36 patients had a right-sided leak, 18 had a left-sided leak, and three had bilateral leaks. It is also interesting to note that most of the post-traumatic leaks were left-sided. Twenty-two patients had a spontaneous onset of the CSF leak, while nine patients had a post-traumatic leak. Five patients were post-surgical: following functional endoscopic sinus surgery (FESS), excision of astrocytoma, meningocele, arachnoid cyst and hemangiopericytoma maxilla and skull base, respectively (Table.1). 

**Table 1:** Etiology of CSF rhinorrhea

**Etiology**	**N**
Spontaneous	22
Traumatic	9
Post-operative FESS	1
Meningocele	1
Arachnoid cyst	1
Astrocytoma	1
Hemangiopericytoma	1

The most common site of leak was the cribriform plate (61%), followed by isolated fovea ethmoidalis, and involvement of both cribriform plate and fovea. One patient had a posterior table of frontal sinus involvement and another had a leak in the lateral recess of the sphenoid sinus (Table.2).

**Table 2:** Location of CSF rhinorrhea

**Location**	**N**
Cribriform plate	22
Fovea ethmoidalis	7
Cribriform plate + fovea ethmoidalis	5
Frontal sinus	1
Sphenoid sinus	1

Two patients had recurrent CSF leak 3 months after the initial endoscopic repair (6%). In the first patient who had a recurrence, the size of the bony defect was 3.5 cm with an intervening sliver of bone. In the second patient, although the size of the leak was only 1.5 cm, there was an associated history of persistent cough which required treatment. Both patients had their site of leak identified in the fovea ethmoidalis on revision surgery.

## Discussion

CSF rhinorrhea occurs as a consequence of breakdown in the dura, arachnoid and a bony skull base defect, resulting in a fistula between the sub-arachnoid space and the nasal cavity.CSF rhinorrhea may occur directly through the anterior cranial fossa or indirectly from the middle or posterior fossa via the Eustachian tube. 

The diagnosis and localization of the leak is crucial for surgical management. The endoscopic examination of the nose is invaluable in confirming the site of leak prior to surgery. Preoperative assessment for the site of leak is performed using a high-resolution CT scan of the paranasal sinuses and anterior skull base. This is currently accepted as the imaging technique of choice. Additional MRI (with T2- weighted sequences) is indicated when parenchymal or meningeal herniation is suspected (2,3).

Banks ^3^ consider intrathecal fluorescein as the most accurate modality for identification of the site of the leak. Intrathecal fluorescein, however, is controversial due to multiple reported complications (5). Seth in his recent series of 103 patients, reported that the lack of intraoperative fluorescein visualization does not rule out the presence of a CSF leak (6). Presutti advocated the easy and safe Valsalva maneuver to be performed by the anesthetist intra-operatively (2). In our study we did not use intrathecal fluorescein for intraoperative confirmation of site of the leak. Instead the Valsalva maneuver was performed where the leak was reconfirmed in all but one patient intra-operatively. 

The size of the defect appears to impact the surgical outcome, with larger defects likely to result in failure of repair and recurrent leaks. Composite osteomucosal or chondromucosal flaps have also been advocated^7,8 ^for repair of defects greater than 3-4 cm. Additionally, co-morbid conditions such as chronic cough may contribute to raised intracranial tension and failure of the repair. 

Presutti, in their 5-year retrospective study of 52 patients with endoscopic closure of CSF leak (2), used a septal mucoper- chondrial graft, with no lumbar drain and fluorescein tests. They reported a success rate of 88.5% on the first attempt. Banks, in their 21-year retrospective study of 193 patients with endoscopic closure using intrathecal fluorescein localization of site of leak and lumbar drain in 73% (3), had an initial success rate of 85–90% and an overall success rate of 98%. Ye (9), in their 10-year retrospective study of 69 patients with no preoperative fluorescein injection, reported a success rate of 89% on the first attempt with an endoscopic multilayer reconstructive technique. Our results of endoscopic CSF rhinorrhea repair revealed a 100% success rate on the first attempt with a recurrence rate of 6%; and 97% success on the second attempt. It is possible that the recurrence was due to multiple sites of leak, which were not recognized during initial surgery. Another possibility is that there were areas of bony defect on the skull base with dural dehiscence which opened upon repair of the area of initial CSF leak. The main contributing factors for failure and recurrence of leak are non-identification of the site, large size of the defect, multiple sites of leak and associated conditions such as chronic cough.

## Conclusion

The most common etiology of CSF rhinorrhea in our study was spontaneous, unlike reports from the West, and the condition is more often seen among women. Better results can be achieved when the following factors are taken into consideration: exact localization of site on MRI/CT prior to surgery, with intraoperative demonstration of the leak by Valsalva maneuver; a three-layer closure technique with fat, fascia, mucosa and fibrin sealant interposition is preferred for small leaks and a meticulous four-layer closure with use of cartilage to give added strength in the repair of large defects.
